# Defensive insect symbiont leads to cascading extinctions and community collapse

**DOI:** 10.1111/ele.12616

**Published:** 2016-06-10

**Authors:** Dirk Sanders, Rachel Kehoe, FJ Frank van Veen, Ailsa McLean, H. Charles J. Godfray, Marcel Dicke, Rieta Gols, Enric Frago

**Affiliations:** ^1^ Centre for Ecology & ConservationCollege of Life and Environmental SciencesUniversity of ExeterPenryn Campus, PenrynCornwallTR10 9FEUK; ^2^ Department of ZoologyUniversity of OxfordSouth Parks RoadOxfordOX1 3PSUK; ^3^ Laboratory of EntomologyWageningen UniversityP.O. Box 16Wageningen6700AAThe Netherlands

**Keywords:** *Acyrthosiphon pisum*, Aphid, *Aphidius ervi*, cascading extinction, defensive symbiosis, endosymbiont, experimental community ecology, *Hamiltonella defensa*, indirect effect, parasitoid

## Abstract

Animals often engage in mutualistic associations with microorganisms that protect them from predation, parasitism or pathogen infection. Studies of these interactions in insects have mostly focussed on the direct effects of symbiont infection on natural enemies without studying community‐wide effects. Here, we explore the effect of a defensive symbiont on population dynamics and species extinctions in an experimental community composed of three aphid species and their associated specialist parasitoids. We found that introducing a bacterial symbiont with a protective (but not a non‐protective) phenotype into one aphid species led to it being able to escape from its natural enemy and increase in density. This changed the relative density of the three aphid species which resulted in the extinction of the two other parasitoid species. Our results show that defensive symbionts can cause extinction cascades in experimental communities and so may play a significant role in the stability of consumer‐herbivore communities in the field.

## Introduction

Virtually all animals are under strong natural selection to avoid predation, parasitism or pathogen infection, and as a consequence, they have evolved a variety of behavioural, mechanical, structural and chemical defences (Evans & Schmidt 1990; Eisner *et al*. 2007). It is increasingly becoming recognised that a further way animals can acquire protection against natural enemies is through association with microbial symbionts (Flórez *et al*. 2015). These defensive symbioses are particularly well studied in herbivorous insects (Hansen & Moran 2014; Oliver *et al*. 2014). Obligate insect microbial symbionts have long been known to be essential for some species because they provide essential nutrients absent in their diets (Barbosa *et al*. 1991; Douglas 2015). The last few decades have seen increasing interest in the evolution, diversity and persistence of facultative associations, and in particular, in those with a defensive role. Facultative defensive symbionts can provide their insect host with increased protection from predators, pathogens and parasitoids (reviewed in Flórez *et al*. 2015). Laboratory experiments with aphid and *Drosophila* populations have shown that the presence of natural enemies can lead to an increase in the frequency of defensive symbionts (Oliver *et al*. 2008; Jaenike & Brekke 2011). In natural populations, the benefits conferred by defensive symbionts can allow their insect hosts to spread spatially (Cockburn *et al*. 2013), and even within the same season, natural enemy pressure can rapidly increase the proportion of individuals carrying defensive microorganisms (Smith *et al*. 2015).

Research on defensive symbionts has tended to focus on their direct effects on the interaction between host and natural enemy (Oliver *et al*. 2010; Frago *et al*. 2012). However, recent advances in insect community ecology have made it increasingly clear that changes in direct interactions between a pair of species can have far‐reaching indirect effects within networks of interacting species (Saterberg *et al*. 2013; Stam *et al*. 2013). Indirect interactions occur when one species affects the dynamics of a second not by a direct trophic or behavioural effect but mediated through the density, behaviour or trait of a third (or more) species. Indirect interactions can be important in promoting species persistence, community stability and ultimately maintaining higher levels of diversity (van Veen *et al*. 2005; Ives & Carpenter 2007; Estes *et al*. 2011). The elimination of indirect interactions can destabilise ecological communities and lead to extinction cascades (Sanders *et al*. 2013, 2015; Saterberg *et al*. 2013). To give an example, community persistence can be enhanced when multiple consumer species specialise on different, potentially competing prey (Vandermeer 1980; Sanders & van Veen 2012). If a particular consumer species is lost or becomes rare, interspecific competition between prey species can increase leading to their extinction, and as a consequence consumer species can also be lost, an extinction cascade (Sanders *et al*. 2013, 2015). Identifying indirect interactions, in this case between the focal consumer, the prey it does not attack, and the other consumers, is the key to understanding community stability. Indirect interactions are likely to be particularly important in maintaining the diversity of insect herbivore communities because they typically support a complex of web of natural enemies, many of which are moderately to highly specialised (van Veen *et al*. 2006). The introduction of a defensive symbiont into a host population can act to remove a specific natural enemy from a community and we hypothesise that this can have effects not only on the host species but also on other members of the community mediated by indirect interactions.

**Figure 1:**
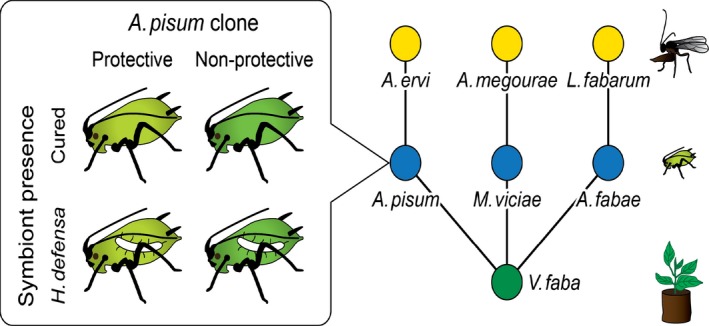
Experimental design. Cages were established with three species of aphids (*Acyrthosiphon pisum*,* Aphis fabae* and *Megoura viciae*) feeding on *Vicia faba*, along with their specialist parasitoids (*Aphidius ervi*,* Lysiphlebus fabarum* and *Aphidius megourae* respectively). The clone and symbiont infection status of *A. pisum* differed between cages: clones originally hosted either a protective symbiont or a non‐protective symbiont, and were either in their natural, infected state or had previously been cured of *Hamiltonella defensa*.

The pea aphid (*Acyrthosiphon pisum*) is a model system for the study of insect symbiosis, and most individuals carry one or two species of facultative symbionts (Oliver *et al*. 2006, 2010; Henry *et al*. 2013). These include the endosymbiont *Hamiltonella defensa*, the first microbe found to have a protective effect against parasitic wasps (Oliver *et al*. 2003, 2005), though subsequent studies have shown that both defensive and non‐defensive strains of this symbiont occur in *A. pisum* (Mclean & Godfray 2015). Clonal lines of asexually reproducing pea aphids can be established in the laboratory, and *H. defensa* can be removed using specific antibiotics or introduced by injection. Here, we study the effect of this symbiont on an aphid–parasitoid community composed of three aphid species (*Acyrthosiphon pisum*,* Aphis fabae* and *Megoura viciae*) and their associated specialist parasitic wasps (*Aphidius ervi*,* Lysiphlebus fabarum* and *Aphidius megourae* respectively). In this community (made up of a particular combination of genotypes), all species are required for long‐term persistence in experimental cage populations (Sanders *et al*. 2013, 2015). We hypothesise that the introduction of a defensive symbiont will destabilise this community and trigger an extinction cascade, and test this with *H. defensa* in *A. pisum*. We established four different types of replicated experimental communities, identical apart from the *A. pisum* (Fig. 1). Two community types included an *A. pisum* clone that naturally hosted a protective form of *H. defensa*; in one community, the aphids were in their natural, infected state but in the other, the symbiont had been removed using antibiotics. The other two communities included a different clone of *A. pisum* which naturally hosted a non‐protective form of the symbiont; in one community, the aphids retained their symbiont and in the other, the symbiont had been removed. We hypothesised that the protective endosymbiont will weaken the interaction between *A. pisum* and its associated parasitoid *A. ervi* leading to higher *A. pisum* densities. This would affect community stability through increased interspecific competition and a reduction in the densities of the other two aphid species which will increase the risk of their extinction or the extinction of their parasitoids. In the communities that included *A. pisum* with the non‐protective symbiont strain, we did not expect *A. pisum* densities to increase or for there to be indirect effects influencing community stability. During the course of the study, we found that cured lineages of the aphid clone that carried the protective symbiont had higher population growth rates than the clone that carried the non‐protective variant. This led us to predict that when comparing communities with the two different symbiont‐free *A. pisum* clones, the lower competitive ability of the non‐protective clone will lead to reduced densities of *A. pisum* and increased extinction of the parasitoid *A. ervi*. At the community level, the loss of *A. pisum* aphids and *A. ervi* parasitoids would also affect community stability through changes in interspecific competition. To understand better how non‐host aphids might affect a parasitoid species’ foraging behaviour and ultimately trigger its extinction, we conducted a behavioural experiment with the parasitoid *A. ervi*. We hypothesised that attacks on its own host, *A. pisum*, will be reduced in the presence of non‐host aphids that altered parasitoid foraging.

## Material and Methods

### Study system

Replicated plant–aphid–parasitoid communities were constructed. They consisted of bean plants (*Vicia faba*, L., var. the Sutton) fed upon by three species of aphid: *Acyrthosiphon pisum* (Harris), *Aphis fabae* (Scopoli) and *Megoura viciae* (Buckton). Each aphid species was attacked by a specialist parasitoid species: *Aphidius ervi* (Haliday), *Lysiphlebus fabarum* (Marshall) and *Aphidius megourae* (Stary) respectively (Fig. 1). In the experiments, two clones of *A. pisum* in which we manipulated the presence of their natural secondary symbiont, *H. defensa*, were employed. Symbiont removal was achieved using a specific antibiotic curing protocol which does not affect the primary symbiont (McLean *et al*. 2011). The first clone was collected on *Medicago sativa* and the strain of *H. defensa* it carries confers strong resistance again the parasitoid *A. ervi*. The second clone was collected on *Ononis spinosa* and carried a strain of *H. defensa* that appears to provide no protection against *A. erv*i (Mclean & Godfray 2015). Below, we shall refer to these *H. defensa* strains as protective and non‐protective. *H. defensa* was absent from the two other aphid species. The *Medicago* strain of *A. pisum* also carried a second facultative symbiont, X‐type, which was not affected by the procedure we used to manipulate *H. defensa* presence. Note that while the two *A. pisum* clones belong to biotypes associated with *Medicago* and *Ononis*, they, like nearly all pea aphid biotypes, flourish on *Vicia faba* which has been described as a ‘universal host’ (Ferrari *et al*. 2008).

### Experiment

The experiment consisted of four treatments with identical species present, but which varied in the clone and symbiont status of *A. pisum*. Two *A. pisum* clones (*Medicago* vs. *Ononis*) with the presence or absence of its natural strain of *H. defensa* were used. Communities were maintained in 47.5 cm^3^ cube gauze cages (BugDorm 44545, Taichung, Taiwan). They were initiated by introducing five wingless adults of the three aphid species spread across four pots (15 cm diameter) each of which contained four 2‐week‐old bean plant seedlings. Two adult mated females of each parasitoid species were added 2 weeks later, and a second pair at week three. This ensured an overlap of parasitoid generations. Each treatment was replicated 10 times in a controlled temperature room at 20 ± 3 °C and a 16/8 h light–dark cycle. To ensure all treatments were exposed to similar conditions, the cages were arranged in ten spatial blocks each containing the four different community types. Beginning 2 weeks after the introduction of the parasitoids, the numbers of aphids and parasitoid mummies on half the plants in each cage was counted once a week. On some occasions, parasitoids were recorded but no hosts. This happened when aphids were very rare (prior to extinction) and by chance none occurred on the half of the plants that were sampled, and because of the natural lag between aphid and parasitoid extinction. Twice a week, the two oldest pots of bean seedlings were replaced by two containing fresh 2‐week‐old seedlings; the old stems from the discarded plants were retained in the cages to avoid loss of insects from the system. Our previous studies had shown that this protocol allows the long‐term persistence of this community of competing aphid species and their natural enemies (Sanders & van Veen 2012; Sanders *et al*. 2013, 2015).

### Behavioural experiment

We measured the impact of non‐host aphids on parasitoid foraging behaviour in *A. ervi*. The upper part of a 3‐week‐old bean plant (including two leaves) was cut off, and the stem inserted upright in 10% agar to maintain freshness. In addition to 20 *A. pisum* aphids, the different treatments contained 20 *M. viciae* aphids, 20 *A. fabae* aphids, or 10 of each aphid species. All aphids were 3–4 days old, and plants with *A. pisum* only were considered as controls. The plants with aphids were placed in a 250‐mL glass beaker, and after 20 min, a mated and experienced female wasp was released inside. The number of attacks on the different aphid species was recorded over a 10‐min period. We considered a parasitoid attack when females exhibited the stereotypical egg laying behaviour in Braconidae aphid parasitoids which consists of extending the abdomen frontally through the legs, and touching the aphid with the abdomen's terminal part. Six wasps that attacked < 5 aphids were excluded from the analyses.

### Statistical analysis

All data were analysed using the open source software R 3.1.3 (R development Core Team). We calculated the initial growth rate of the different aphid populations in the first 30 days of the experiment before the emergence of the first generation of parasitoids (growth rate = (*N*(30) − *N*(0))/30 where *N*(*x*) is population density on day *x*). We used anova to test the effects of clone and symbiont presence on initial population growth rate. The impact of these treatments on aphid and parasitoid population dynamics were analysed by building independent linear mixed effects models for each clonal lineage with symbiont presence as fixed factor. To account for systematic trends over time, week, and week squared were included as covariates while cage nested in block was included as a random factor. Because the residuals of the models showed significant temporal autocorrelation, a first‐order autoregressive term was included. Model simplification was carried out by sequentially removing non‐significant interactions within the function *lme* from the package *nlme* (Pinheiro *et al*. 2015). We additionally analysed aphid and parasitoid dynamics in all four treatments at the same time by building similar models, but including clonal lineage, symbiont and their interaction as fixed factors. Percent variance explained by fixed factors in mixed models was estimated as pseudo‐*R* ^2^ values using the function *r.squaredGLMM* from the package *MuMIn* (Barton 2016). Persistence of the different species in the community was analysed with nonparametric Kaplan–Meier survival analysis using the function *survdiff* in the package *survival* (Harrington & Fleming 1982). Species that persisted in cages until the end of the experiment were treated as censored data.

Aphid relative abundance was analysed using generalised linear mixed models assuming a binomial error distribution and using the logit link function. The dependent variable was the bivariate variable containing ‘abundance of aphid species *i*’ and ‘total aphid abundance – abundance of aphid species *i*’, where ‘*i*’ can be the abundance of *A. fabae*,* M. viciae*, or *A. pisum*. Symbiont or clone treatments were included as fixed factors, and week and week squared as covariates. Week squared was included in the models as a covariate to account for systematic nonlinear trends over time and to increase model fit. Block and replicate nested in block were treated as random factors and we also included a random slope for the week effect per replicate. Since a degree of over‐dispersion was detected, an observation‐level random factor was also included (Browne *et al*. 2005). The analysis used the function *glmer* from the package *lme4* (Bates *et al*. 2014). Model simplification was carried out by sequentially removing non‐significant interactions. To obtain 95% credible intervals for the model predictions, we used Bayesian methods to draw a random sample of 1000 values from the posterior distribution of the model parameters. This was done employing the function *sim* from the package *arm* (Gelman & Yu‐Sung 2015). From these 1000 model parameter sets, predicted values were calculated and their 2.5 and 97.5% quantiles were used as lower and upper limits of the 95% credible intervals. Parasitoid attacks in the behavioural experiment were analysed with generalised linear models assuming a quasi‐poisson error distribution.

## Results

### The effect of the protective symbiont on *A. pisum* and its parasitoid

**Figure 2:**
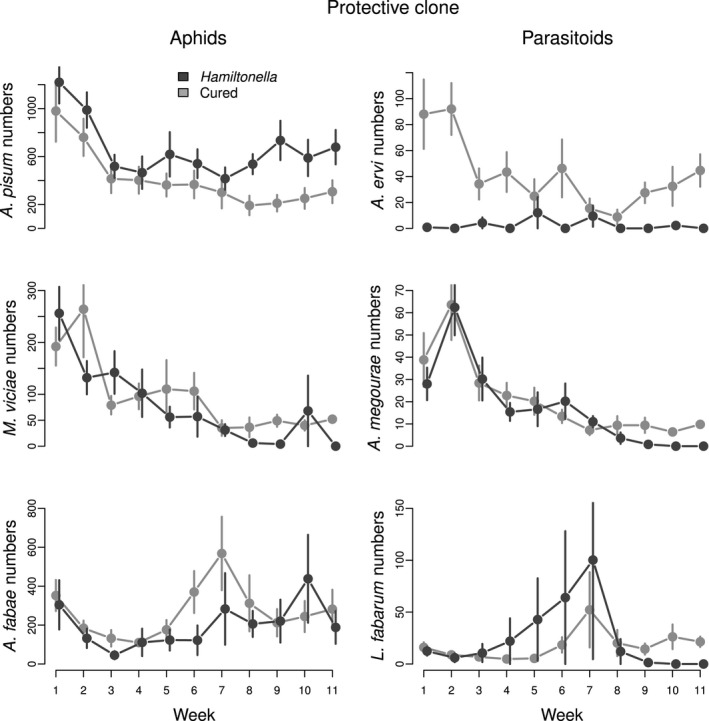
Long‐term dynamics of the community where the symbiont status was manipulated in the *Acyrthosiphon pisum* aphid clone carrying the protective *Hamiltonella defensa* strain. Dark grey lines and bars represent species abundance (± SE) in replicates where the symbiont was present, and light grey lines and bars represent those where the symbiont was absent.

We predicted that the presence of the protective symbiont would lead to higher *A. pisum* densities and negatively affect its parasitoid, *A. ervi*. The results supported this prediction. The mean cumulative density of *A. pisum* was 1.6 times greater in replicates with the symbiont compared to without (*F*_1,9_ = 6.93, *P = *0.027), while the mean cumulative density of *A. ervi* was over 16 times less (*F*_1,9_ = 98.32, *P < *0.001; Table S1; Fig. 2). The symbiont effect on *A. ervi* numbers varied through time as revealed by a significant interaction between time and treatment (*F*_1,197_ = 5.89, *P = *0.016; Table S1; Fig. 2). In replicates with the symbiont, the parasitoids always had low densities, whereas without the symbiont, an initial peak was followed by a decrease in density after the third week (*F*_1,197_ = 5.89, *P = *0.016; Table S1; Fig. 2). The relative abundance of *A. pisum* was slightly higher in those cages with the symbiont, although this was marginally non‐significant (*Z* = 1.89, *P* = 0.058; Table S2; Fig. 4). *A. pisum* aphids became extinct in 10% of the cages irrespective of the presence of the symbiont ($\chi_{2}^{2}$ < 0.01, *P = *0.970; Table S1; Fig. 5: panel A1). The parasitoid, however, went extinct in all cages with the symbiont present while it persisted in all communities without the symbiont, a significant difference ($\chi_{2}^{2}$ = 22.00, *P < *0.001; Table S1; Fig. 2: panel P1). A detailed description of the statistical analyses is provided in the online supplementary material (Tables S1 and S2).

### The effect of the protective symbiont at the community level

We expected that the higher densities of *A. pisum* brought about by the presence of the protective symbiont would, through greater resource competition, negatively affect the two other aphids, *M. viciae* or *A. fabae*, and that this would in turn reduce the densities and persistence of their specific parasitoids. The cumulative mean numbers of *M. viciae* and *A. fabae* did not differ between the two treatments (*M. viciae: F*_1,9_ = 4.79, *P = *0.057; *A. fabae: F*_1,9_ = 2.90, *P = *0.123; Table S1; Fig. 2). The effect of the symbiont on *M. viciae* numbers became stronger over time (there was a significant interaction between time and the symbiont treatment, *F*_1,197_ = 11.11, *P < *0.001; Table S1; Fig. 2). Despite the absence of differences in total abundance, *M. viciae* relative abundance significantly declined from week six onwards, to < 1% in treatments with the symbiont compared to 5–7% without the symbiont (*Z = *3.43, *P < *0.001; Table S2; Fig. 4). Similarly, *A. fabae* relative abundance declined from 27 to 0.9% when the symbiont was present, a significant difference (*Z = *−2.24, *P = *0.025; Table S2; Fig. 4). Towards the end of the experiment, *A. megourae* parasitoid numbers were lower when the symbiont was present (symbiont effect: *F*_1,9_ = 5.22, *P = *0.048; symbiont × week effect: *F*_1,197_ = 8.07, *P = *0.005; Table S1; Fig. 2). The presence of the symbiont did not affect the numbers of the parasitoid *L. fabarum* (*F*_1,9_ = 2.26, *P = *0.137; Table S1; Fig. 2). There was an effect of the symbiont on the persistence of *M. viciae* ($\chi_{2}^{2}$ = 7.09, *P = *0.008), but not of *A. fabae* ($\chi_{2}^{2}$ = 3.45, *P = *0.063; Table S1; Fig. 5: panels A2 & A3)*. M. viciae* became extinct in all cages when the symbiont was present, but only in 30% of replicates without the symbiont. The greatest indirect effect of the presence of the symbiont was on the persistence of the specific parasitoids *A. megourae* and *L. fabarum* which became extinct in all cages when the symbiont was present, but persisted in all communities when the symbiont was absent (*A. megourae* $\chi_{2}^{2}$ = 21.40, *P* < 0.001; *L. fabarum* $\chi_{2}^{2}$ = 20.30, *P* < 0.001; Table S1; Fig. 5: panels P2 & P3).

### The effect of the non‐protective symbiont on *A. pisum* and its parasitoid

**Figure 3:**
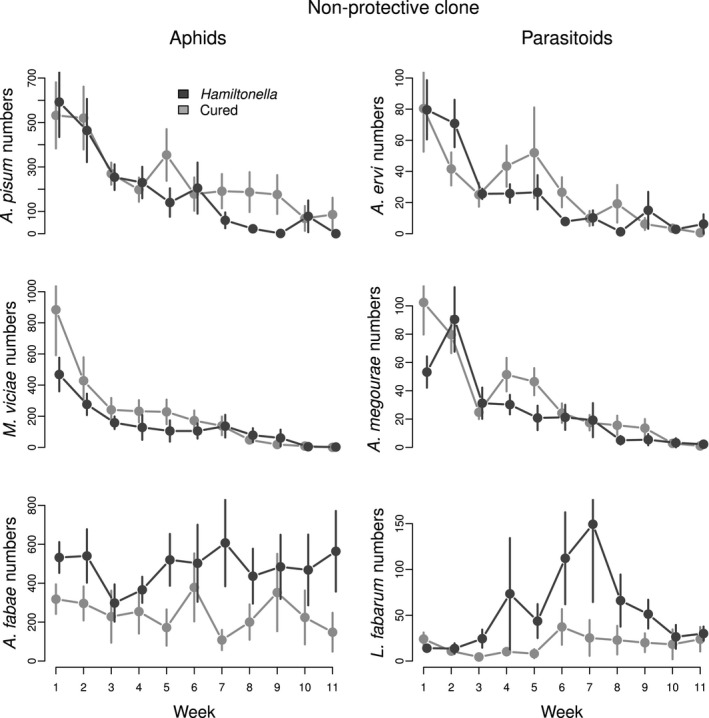
Long‐term dynamics of the community where the symbiont status was manipulated in the *Acyrthosiphon pisum* aphid clone carrying the non‐protective *Hamiltonella defensa* strain. Dark grey lines and bars represent species abundance (± SE) in replicates where the symbiont was present, and light grey lines and bars represent those where the symbiont was absent.

**Figure 4:**
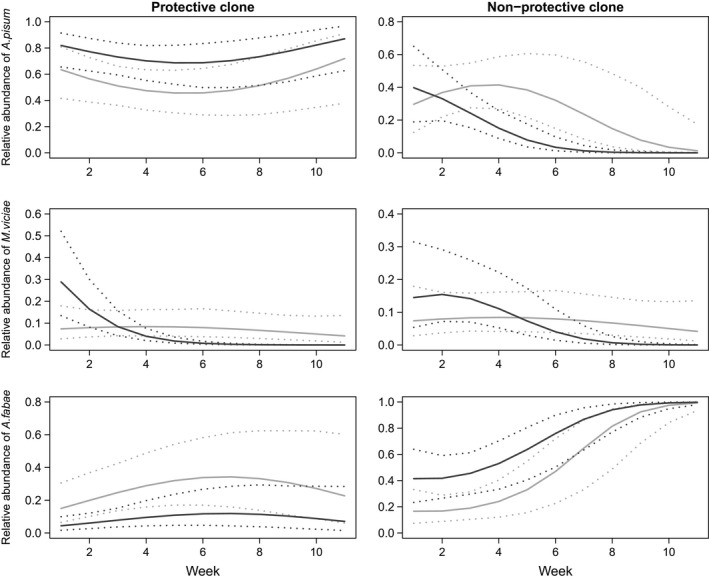
Relative aphid abundance (solid line) and 95% confidence intervals (dotted line) for model predictions in communities where the aphid clone (collected on *Medicago* and protected, or collected on *Ononis* and non‐protected) and the symbiont *Hamiltonella defensa* (present or absent) were manipulated in *Acyrthosiphon pisum* aphids. Communities without the symbiont are represented with light grey lines, and those with the symbiont are represented with dark grey lines.

**Figure 5:**
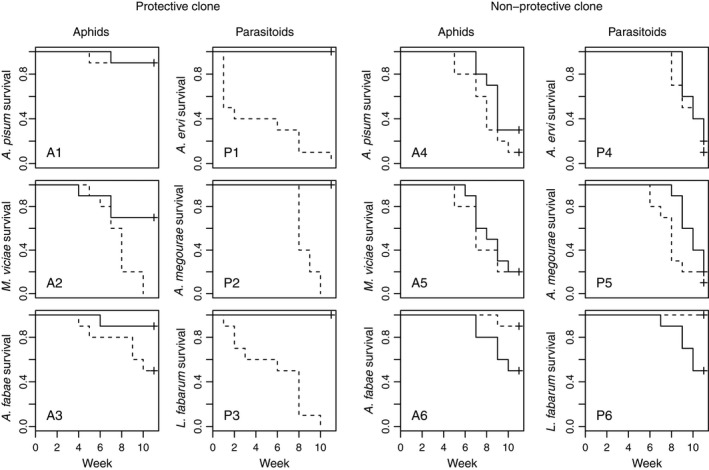
Persistence of the six species in communities where the aphid clone (collected on *Medicago* and protected, and collected on *Ononis* and non‐protected) and the symbiont *Hamiltonella defensa* (present or absent) were manipulated in *Acyrthosiphon pisum* aphids. The *y*‐axis represents the proportion of microcosm cages in which each species survived. Dashed lines represent communities with the symbiont present, and solid lines represent those without the symbiont.

We predicted that the presence of the non‐protective symbiont would not lead to higher *A. pisum* aphid densities (and might even reduce their numbers if symbiont carriage was costly), and that it would have no effect on *A. ervi* dynamics. *A. pisum* densities were slightly lower in the presence of the symbiont, but overall there were no significant differences in *A. pisum* or *A. ervi* densities, and their interaction with time (*A. pisum: F*_1,9_ = 3.51, *P = *0.094*; A. ervi: F*_1,9_ = 0.20, *P = *0.669; Table S1; Fig. 3). The relative abundance of *A. pisum* was not affected by the symbiont, but the symbiont had a significant effect on the time course of relative *A. pisum* abundances (symbiont effect: *Z = *1.21, *P = *0.226; symbiont × week effect: *Z = *−2.64, *P = *0.008; Table S2; Fig. 4). By the end of the experiment, *A. pisum* relative abundance was very low in both treatments, but during week four to eight, abundances were lower when the symbiont was present (Fig. 4). Aphid and parasitoid persistence were also unaffected by the symbiont (*A. pisum:* $\chi_{2}^{2}$ = 2.37, *P = *0.123; *A. ervi:* $\chi_{2}^{2}$ = 0.49, *P = *0.486; Table S1; Fig. 5: panels A4 & P4).

### The effect of the non‐protective symbiont at the community level

We did not expect to see any community consequences of the presence of the non‐protective symbiont in *A. pisum* aphids. The presence of the symbiont did not affect *M. viciae* and *A. megourae* densities (*M. viciae: F*_1,9_ = 1.68, *P = *0.227; *A. megourae: F*_1,9_ = 2.93, *P = *0.121; Table S1; Fig. 3) or their persistence (*M. viciae:* $\chi_{2}^{2}$ = 0.16, *P = *0.688; *A. megourae:* $\chi_{2}^{2}$ = 3.10, *P = *0.078; Table S1; Fig. 5: panels A5 and P5), but the relative abundance of *M. viciae* was lower when the symbiont was present, a marginally significant difference (*Z = *−1.96, *P = *0.049; Table S2; Fig. 4). However, there was an effect of symbiont presence on the other aphid–parasitoid pair. *A. fabae* numbers were on average 1.9 times higher when the symbiont was present (*F*_1,9_ = 6.62, *P = *0.030; Table S1; Fig. 3) and while this species became the dominant aphid in both treatments, this occurred more quickly in the symbiont replicates (*Z = *2.38, *P = *0.017; Table S2; Fig. 4). *A. fabae* became extinct in 50% of the cages when the symbiont was absent, but persisted in 90% of them when the symbiont was present, a marginally non‐significant difference ($\chi_{2}^{2}$ = 3.76, *P = *0.053; Table S1; Fig. 5: panel A6). Differences in the mean densities of the parasitoid *L. fabarum* were marginally significant (*F*_1,9_ = 5.44, *P = *0.045; Table S1; Fig. 3). This parasitoid never went extinct in the presence of the symbiont, but it was lost in half the replicates where the symbiont was absent ($\chi_{2}^{2}$ = 6.34, *P = *0.019; Table S1; Fig. 5: panel P6).

### The effect of *A. pisum* clonal differences on *A. pisum* and its parasitoid

The two pea aphid clones used in the experiment were chosen because they were the natural hosts of the protective and non‐protective symbionts strains, rather than to test any *a priori* hypotheses. During the establishment of our experimental communities, it was noted that in the absence of the symbiont, the *A. pisum* clone that hosted the protective symbiont had a significantly higher initial population growth rate compared to the clone that carried the non‐protective one (15.9 ± 2.3 (mean ± SE) vs. 8.0 ± 1.6; *F*_1,36_ = 8.05, *P* = 0.007). Although differences in growth rate were not affected by the presence of the symbiont (effects of symbiont: *F*_1,36_ = 0.62, *P* = 0.434; clone × symbiont interaction: *F*_1,36_ = 0.23, *P* = 0.638), we analysed insect dynamics only in the replicates without symbionts to avoid any possible confounding effects of the bacteria. The difference in initial population growth rate was reflected in significantly lower densities of *A. pisum* and *A. ervi* for the slower growing clone (*A. pisum*:* F*_1,9_ = 8.09, *P = *0.019*; A. ervi: F*_1,9_ = 6.23, *P = *0.034; Table S1; Figs. 3 and 4). Relative *A. pisum* abundance was also lower for the slower growing clone (27 vs. 44%, *Z = −*2.38, *P = *0.017; Table S2; Fig. S4), and both *A. pisum* and its associated parasitoid had significantly greater probabilities of extinction (*A. pisum:* $\chi_{2}^{2}$ = 6.33, *P = *0.012; *A. ervi:* $\chi_{2}^{2}$ = 13.22, *P < *0.001; Table S1; Fig. 5: solid lines in panels A1, A4, P1 & P4). To assess the effect of the symbiont on the two different *A. pisum* clones, we also analysed aphid and parasitoid dynamics in all four treatments simultaneously to test the interaction term. Aphid clone had a significant effect on *A. pisum* cumulative numbers (*F*_1,27_ = 58.06, *P < *0.001), and the symbiont effect varied between the two aphid clones used (symbiont × clone interaction: *F*_1,27_ = 10.89, *P < *0.001; Table S3). Mean cumulative numbers of the parasitoid *A. ervi* were significantly affected by the presence of the symbiont (*F*_1,27_ = 84.85, *P < *0.001), an effect that varied between aphid clones (symbiont × clone interaction: *F*_1,27_ = 79.07, *P < *0.001; Table S3). In a mixed model with all fixed factors (week, week squared, symbiont presence and aphid clone), the percent variance explained by the symbiont and aphid clone was, respectively, 0.84 and 47.69% in *A. pisum* models, and 40.24 and 4.17% in *A. ervi* models.

### The effect of *A. pisum* clonal differences at the community level

Given the observed difference in *A. pisum* clonal performance, we predicted that the other two aphid species and their parasitoids would be at an advantage in communities with the slower growing clone. There were no differences in the cumulative numbers of *M. viciae* or *A. fabae* aphids, or their associated parasitoids *A. megourae* and *L. fabarum*, in communities with the two *A. pisum* clones (*M. viciae: F*_1,9_ = 2.30, *P = *0.164*; A. fabae: F*_1,9_ = 1.03, *P = *0.336; *A. megoura*e: *F*_1,9_ = 2.60, *P = *0.141; *L. fabarum: F*_1,9_ = 0.14, *P = *0.722*;* Table S1; Figs. 2 and 3). At the beginning of the experiment, the relative density o*f M. vici*ae was significantly larger in treatments with the slower growing *A. pisum* clone, although densities were similar towards the end of the experiment (clone × week interaction *Z = *2.48, *P* = 0.0133; Table S2; Fig. S4). The relative density of *A. fabae* was not affected by *A. pisum* clone (*Z = −*0.85, *P = *0.393; Table S2; Fig. S4) nor was the probability of aphid persistence (*M. viciae:* $\chi_{2}^{2}$ = 3.55, *P = *0.061; *A. fabae:* $\chi_{2}^{2}$ = 3.11, *P = *0.078; Table S1; Fig. 5: solid lines in panels A2, A3, A5 & A6). There was, however, a difference in the probability of parasitoid persistence; both *A. megourae* and *L. fabarum* where significantly more likely to go extinct in cages with the slower growing aphid clone (*A. megourae:* $\chi_{2}^{2}$ = 13.11, *P < *0.001; *L. fabarum:* $\chi_{2}^{2}$ = 6.33, *P = *0.012; Table S1; Fig. 5: solid lines in panels P2, P3, P5 & P6). This was the opposite of what we expected. To test the interaction between the symbiont and *A. pisum* clone, aphid and parasitoid dynamics were analysed within a single factorial framework. Mean cumulative numbers of *M. viciae* aphids and their associated parasitoid *A. megourae* were significantly affected by the symbiont in *A. pisum* aphids (*M. viciae: F*_1,27_ = 9.70, *P = *0.004*; A. megoura*e: *F*_1,27_ = 9.89, *P = *0.004), but not by *A. pisum* clone (*M. viciae: F*_1,27_ = 0.11, *P = *0.919; *A. megoura*e: *F*_1,27_ = 0.54, *P = *0.470; Table S3). The symbiont effect was consistent between the two clones as revealed by the non‐significance of the interaction terms, which were removed from the models. Mean cumulative numbers of the aphid *A. fabae* and its associated parasitoid *L. fabarum* were not affected by the symbiont in *A. pisum* aphids (*A. fabae: F*_1,27_ = 0.18, *P = *0.677*; L. fabarum*:* F*_1,27_ = 0.30, *P = *0.587; Table S3). Mean cumulative numbers of the aphid *A. fabae* were not affected by *A. pisum* clone (*F*_1,27_ = 1.61, *P = *0.215), but cumulative numbers of parasitoid *L. fabarum* were (*F*_1,27_ = 9.08, *P = *0.006; Table S3). For these two species, the symbiont effect varied between the two *A. pisum* clones (symbiont × clone interaction in *A. fabae: F*_1,27_ = 15.54, *P < *0.001*; L. fabarum*:* F*_1,27_ = 14.76, *P < *0.001; Table S3). In a mixed model with all fixed factors (week, week squared, symbiont presence and aphid clone), the percent variance explained by the symbiont and the aphid clone was, respectively, 4.28 and 3.61% for *M. viciae* models, 15.80 and 44.06% for *A. fabae* models, 4.40 and 2.11% for *A. megourae* models, and 4.01 and 92.24% for *L. fabarum* models.

### Behavioural experiment

Contrary to our prediction, the number of *A. pisum* hosts attacked by *A. ervi* was not affected by the presence of non‐host aphids ($\chi_{2}^{2}$ = 3.43, *P = *0.329, Fig. S5). However, *A. ervi* attacked non‐hosts, particularly *M. viciae* aphids. We analysed *A. ervi* attacks excluding control plants that harboured exclusively *A. pisum* aphids, and found that in the presence of the non‐host *A. megourae*, the parasitoid attacked this host more often than its own host (treatment effect: $\chi_{2}^{2}$ = 4.34, *P = *0.114; non‐hosts effect: $\chi_{2}^{2}$ = 0.51, *P = *0.473; interaction term: $\chi_{2}^{2}$ = 10.65, *P = *0.005; Fig. S5).

## Discussion

Our study shows that manipulation of an endosymbiotic bacterium in an aphid species can affect the long‐term dynamics of an experimental community consisting of three aphid species feeding on the same resource and their associated specialist parasitoid wasps. We manipulated the presence of the endosymbiotic bacterium *H. defensa*, in a pea aphid, *A. pisum*, clone where it confers resistance against the parasitoid *A. ervi*. This defensive phenotype led to higher *A. pisum* densities and the exclusion of its specialist parasitoid which became extinct in all experimental communities. Manipulating the bacterial strain that provided no protection against *A. ervi* did not significantly affect the density of *A. pisum* aphids and their associated parasitoids. As demonstrated previously for both aphids and *Drosophila*, the protective effect of a defensive symbiont commonly leads to higher host abundances in the presence of natural enemies (Oliver *et al*. 2008; Jaenike & Brekke 2011), and we show here that for a particular combination of aphid genotypes and symbiont strains, this protection also occurs in a more complex community with potentially strong interspecific competition at the herbivore trophic level.

The presence of the defensive symbiont in *A. pisum* also affected the dynamics and persistence of other species in the community. The larger numbers of *A. pisum* aphids when the symbiont was present did not lead to a reduction in the absolute density of the other two aphids, *M. viciae* and *A. fabae*, but it did reduce their relative abundance. More dramatically, their two parasitoids *A. megourae* and *L. fabarum* became extinct in all replicates when the protective symbiont was present, but survived in all replicates when it was absent. An explanation for this is suggested by previous work which has shown that the abundance of related non‐host species can affect the efficiency of parasitoids searching for their specific host species (reviewed by van Veen & Godfray 2012). For example, by combining experimental microcosm experiments (involving the aphids *A. pisum*,* M. viciae* and the parasitoid *A. ervi*) with population modelling, van Veen *et al*. (2005) demonstrated that in the presence of non‐host *M. viciae* the parasitoid *A. ervi* had a lower per‐capita attack rate (a form of density‐dependent interference). Increasing the density and diversity of non‐host aphids in the environment has been shown to markedly reduce foraging efficiency in the parasitoids *A. megourae* and *L. fabarum* (Kehoe *et al*., 2016). In the wasp behaviour experiment, we have also found that non‐hosts aphids alter parasitoid foraging. In the presence of the non‐host aphid *M. viciae*, the parasitoid *A. ervi* attacks this species more often than its own host *A. pisum*. In this experiment, wasps were tested for 10 minutes and in a simple scenario, and it is likely that in a more complex community and over the parasitoid's lifespan, these interactions will reduce foraging efficiency. We therefore hypothesise that in the current experiment, the decreased relative frequency of their hosts led to parasitoid wasps spending more time examining, rejecting and attacking unsuitable hosts, so that their searching efficiency and hence reproductive rate declined to a level at which the population could not sustain itself and extinction ensued. An important question for further research is to find out whether such behaviours only occur in confined conditions such as laboratory cages or whether they are relevant in the field.

The density of *A. pisum* thus affects the interaction between *A. megourae* and *M. viciae*, and *L. fabarum* and *A. fabae*, which are indirect effects since no direct resource–consumer (trophic) relationships are involved. The different parasitoid species can be considered to be connected by positive indirect interactions and the loss of one leads to an extinction cascade. A related example of the consequences of the loss of positive indirect interactions has recently been demonstrated in similar experimental aphid communities. Sanders *et al*. (2013) found that removal of one parasitoid species released its host from top‐down control, and triggered the extinction of other indirectly linked parasitoid species. Compared to that study, we found extinction cascades were triggered earlier and in a larger proportion of replicates. A potential explanation for this is that Sanders *et al*. (2013) manipulated the aphid–parasitoid interaction by removing parasitised aphids (mummies) and this reduced parasitoid populations. In our study, however, the protective effect of the symbiont reduced the population growth of *A. ervi* parasitoids, but at the same time prevented the death of the attacked aphids.

We studied the effect of the symbiont on aphid–parasitoid communities in two different *A. pisum* clones: one naturally infected with a *H. defensa* strain that confers on its host a high level of parasitoid protection and the other with no known effect on parasitic wasps (Mclean & Godfray 2015). Contrary to our expectations, the presence of a non‐protective symbiont affected the density of *A. fabae* aphids and its associated parasitoid *L. fabarum*, and in some cases aphid and parasitoid extinctions occurred, though the differences were not significant. Interpreting these results is complicated by differences in the intrinsic growth rates of the two aphid clones in the absence of symbiont. Although not an initial goal of our experiment, this led us to predict that extinction cascades would be triggered in communities with the faster growing clone. In fact, we found the opposite, suggesting that extinction cascades can be triggered not only when *A. pisum* comes to dominate the community but also when this species gets outcompeted by *M. viciae* and *A. fabarum*. Although our study was limited to two different *A. pisum* clones, these results also suggest that not all *A. pisum* genotypes facilitate the long‐term stability of the community, and future work is therefore needed to unveil which particular traits promote stabilising positive indirect interactions. These traits might be influenced by the genotype of the herbivore or its symbiont composition, and might affect the insect susceptibility to natural enemies or traits related to plant exploitation. Herbivory can result in species‐specific changes in plant morphology and physiology that through plant‐mediated indirect effects have cascading consequences for other organisms in the community (Stam *et al*. 2013). Long‐term community experiments can help us understand how indirect interactions involving higher or lower trophic levels modulate interactions among herbivorous species and ultimately affect the stability of terrestrial communities. At the evolutionary level, although aphid colonies are often composed of a single clonal lineage (Vantaux *et al*. 2011), aphids have also been used to show that natural enemy pressure rapidly selects for specific genotypes (Turcotte *et al*. 2011). It would be very interesting to explore evolutionary processes in more complex communities such as the one described here.

Understanding the factors that promote stability and diversity in natural communities is a topic of great relevance at a time when human activities threaten many natural ecosystems (Barnosky *et al*. 2012; Cardinale *et al*. 2012). Our work reinforces the idea that direct and indirect interactions involving consumers and their prey or hosts are important in maintaining diversity in insect communities, and reveals that facultative insect symbionts can modulate the strength of these interactions in important ways. So far, little attention has been paid to the role of insect symbionts in this context, although we believe their consequences can be far‐reaching. There are several examples of facultative symbionts in herbivorous insects that enable their hosts to spread geographically, either through the effects they have on their host's food‐plant utilisation or their susceptibility to natural enemies (reviewed by Frago *et al*. 2012). For example, a genotype of the whitefly *Bemisia tabaci* is spreading around the globe partly due to a mutualism with a virus, which suppresses host‐plant resistance (Li *et al*. 2014). Bark and ambrosia beetles can also become more serious pests by acquiring novel fungal associates that allow them to switch from attacking dead to live trees (Hulcr & Dunn 2011). In *Drosophila neotestacea*, the acquisition of a defensive endosymbiont in the genus *Spiroplasma* provided protection from a parasitic nematode and allowed certain matrilines to spread across central Canada (Cockburn *et al*. 2013).

With the proviso that our experiments took place in population cages and not in the field, and that a single protective strain of *H. defensa* was tested, our study shows that microbial symbionts can influence direct and indirect interactions between species and can thus trigger extinction cascades. Further work is needed in more natural situations to explore this phenomenon, especially to investigate the costs of symbiont carriage in the field and the complexities that may occur in communities containing many more hosts, natural enemies and symbionts than we have studied here. Thanks to the revolution in molecular biological techniques, the last two decades have seen a huge growth in our knowledge of the diversity of insect‐associated microorganisms (Hansen & Moran 2014), but we are only beginning to explore the effects they may have at the community level. A deeper understanding of these effects will provide new insights into the structure and function of one of the most diverse types of community in terrestrial ecosystems, and into the forces that maintain diversity and the ecosystem services diversity provides (Hooper *et al*. 2012).

## Author Contributions

DS and EF designed the study; DS and RK performed the research; AM created and characterised pea aphid lines; DS and EF analysed data; DS, FJFvV, HCJG and EF wrote the manuscript and all authors contributed to discussions and revisions.
