# Supplementary material for: Defensive insect symbiont leads to cascading extinctions and community collapse
Source: Ecol Lett. 2016 Jun 10;19(7):789–99. doi: 10.1111/ele.12616 (PMC4949664; doi:10.1111/ele.12616)
Supplement: Supplementary file 1 [file ELE-19-789-s001.pdf]

Defensive insect symbiont leads to cascading extinctions and community collapse

Dirk Sanders, Rachel Kehoe, Frank Van Veen, Ailsa McLean, H. Charles J. Godfray, Marcel Dicke, Rieta Gols, Enric Frago

Table and figure legends (Supporting information)

Table S1. Mixed effects model analyses, and survival analyses on aphid and parasitoids in communities where the aphid clone (collected on *Medicago* and protected, or collected on *Ononis* and non-protected) and the symbiont *Hamiltonella defensa* (present or absent) were manipulated in *Acyrthosiphon pisum* aphids. Analyses test symbiont effects in both *A. pisum* clones independently, and clone effects in replicates without symbionts. * d.f. are 1, 197 in *M. viciae* models, ** d.f. are 1, 198 in *L. fabarum* models.

Table S2. Mixed effects model analyses on aphid relative abundances in communities where the aphid clone (collected on *Medicago* and protected, or collected on *Ononis* and non-protected) and the symbiont *Hamiltonella defensa* (present or absent) were manipulated in *Acyrthosiphon pisum* aphids.

Table S3. Mixed effects model analyses on aphid and parasitoids in communities where the aphid clone (collected on *Medicago* and protected, or collected on *Ononis* and non-protected) and the symbiont *Hamiltonella defensa* (present or absent) were manipulated in *Acyrthosiphon pisum* aphids. Analyses test all four treatments simultaneously (i.e. symbiont and clone effects, and their interaction). * d.f. are 1, 28 in *M. viciae* model in clone or symbiont parameters, ** d.f. are 1,397 for *A. ervi* in week parameters.

Figure S4. Relative aphid abundance (solid line) and 95% confidence intervals (dotted line) for model predictions in replicates without symbionts where the aphid clone (collected on *Medicago* and fast-growing, or collected on *Ononis* and slow-growing) was manipulated in *Acyrthosiphon pisum* aphids. Communities with the slow-growing clone are represented with light grey lines, and those with the fast-growing clone are represented with dark grey lines.

Figure S5. Parasitoid attacks on host and non-host aphids. Mean number (±SE) of *Aphidius ervi* attacks on *Acyrthosiphon pisum* host aphids (dark grey bars), and on *Megoura viciae* and *Aphis fabae* non-host aphids (light grey bars). Treatments represent *A. pisum* aphids alone, or with one or both of the non-host aphids.

Table S1

Protective clone

Parameters		*Acyrthosiphon pisum*		*Megoura viciae*		*Aphis fabae*	
Aphids	d.f. *	F-value	p	F-value	p	F-value	p
Intercept	1, 198	**318.08**	**<.0001**	**103.59**	**<.0001**	**97.45**	**<.0001**
Symbiont	1, 9	**6.93**	**0.027**	4.79	0.057	2.90	0.123
Week	1, 198	**19.00**	**<.0001**	**108.45**	**<.0001**	0.66	0.418
Week 2	1, 198	**17.09**	**<.0001**	**13.34**	**<.0001**	2.73	0.100
Symbiont x Week	1, 197			**11.11**	**<.0001**		

Parasitoids	d.f. **	*Aphidius ervi*		*Aphidius megourae*		*Lysiphlebus fabarum*	
Intercept	1, 197	**125.72**	**<.0001**	**125.91**	**<.0001**	**23.29**	**<.0001**
Symbiont	1, 9	**98.35**	**<.0001**	**5.22**	**0.048**	2.66	0.137
Week	1, 197	**6.97**	**0.009**	**138.67**	**<.0001**	0.17	0.681
Week 2	1, 197	**8.83**	**0.003**	2.14	0.145	0.12	0.732
Symbiont x Week	1, 197	**5.89**	**0.016**	**8.07**	**0.005**		

Non-protective clone

Parameters		*Acyrthosiphon pisum*		*Megoura viciae*		*Aphis fabae*	
Aphids	d.f.	F-value	p	F-value	p	F-value	p
Intercept	1, 198	**197.66**	**<.0001**	**96.10**	**<.0001**	**96.65**	**<.0001**
Symbiont	1, 9	3.51	0.094	1.68	0.227	**6.62**	**0.030**
Week	1, 198	**84.90**	**<.0001**	**141.69**	**<.0001**	**4.53**	**0.035**
Week 2	1, 198	1.45	0.229	**4.25**	**0.041**	0.48	0.488

Parasitoids	d.f.	*Aphidius ervi*		*Aphidius megourae*		*Lysiphlebus fabarum*	
Intercept	1, 198	**154.19**	**<.0001**	**117.02**	**<.0001**	**70.88**	**<.0001**
Symbiont	1, 9	0.20	0.669	2.93	0.121	**5.44**	**0.045**
Week	1, 198	**142.01**	**<.0001**	**213.83**	**<.0001**	0.47	0.493
Week 2	1, 198	2.79	0.097	1.51	0.221	1.11	0.293

Clonal lineage (cured lines)

Parameters		*Acyrtosiphon pisum*		*Megoura viciae*		*Aphis fabae*	
Aphids	d.f. *	F-value	p	F-value	p	F-value	p
Intercept	1, 198	**217.26**	**<.0001**	**100.22**	**<.0001**	**106.05**	**<.0001**
Clone	1, 9	**8.09**	**0.019**	2.30	0.164	1.03	0.336
Week	1, 198	**34.22**	**<.0001**	**109.83**	**<.0001**	3.34	0.069
Week 2	1, 198	3.91	0.050	**6.44**	**0.012**	0.43	0.515
Symbiont x Week	1, 197			**30.20**	**<.0001**		

Parasitoids	d.f. **	*Aphidius ervi*		*Aphidius megourae*		*Lysiphlebus fabarum*	
Intercept	1, 197	**217.11**	**<.0001**	**134.46**	**<.0001**	**58.99**	**<.0001**
Clone	1, 9	**6.23**	**0.034**	2.60	0.141	0.14	0.722
Week	1, 197	**39.78**	**<.0001**	**140.21**	**<.0001**	0.79	0.377
Week 2	1, 197	**7.43**	**0.007**	**3.98**	**0.047**	1.81	0.181
Symbiont x Week	1, 197	**5.03**	**0.026**	**18.07**	**<.0001**		

Survival

Survival		*Acyrthosiphon pisum*		*Megoura viciae*		*Aphis fabae*	
Parameters	d.f.	X^2	p	X^2	p	X^2	p
Symbiont (protective clone)	1	0.00	0.970	**7.09**	**0.008**	3.45	0.063
Symbiont (non-protective)	1	2.37	0.123	0.16	0.688	3.76	0.053
Clonal lineage (cured lines)	1	**6.33**	**0.012**	3.55	0.061	3.11	0.078

		Aphidius ervi		*Aphidius megourae*		*Lysiphlebus fabarum*	
	d.f.	X^2	p	X^2	p	X^2	p
Symbiont (protective clone)	1	**22.00**	**<.0001**	**21.40**	**<.0001**	**20.30**	**<.0001**
Symbiont (non-protective)	1	0.49	0.486	3.10	0.078	**6.34**	**0.019**
Clonal lineage (cured lines)	1	**13.22**	**<.0001**	**13.11**	**<.0001**	**6.33**	**0.012**

Table S2

Protective clone

Parameters	*Acyrthosiphon pisum*				*Megoura viciae*				*Aphis fabae*			
	Estimate	Std	z-value	p	Estimate	Std	z-value	p	Estimate	Std	z-value	p
Intercept	0.91	0.61	1.51	0.132	**-2.64**	**0.69**	**-3.84**	**0.000**	**-2.14**	**0.61**	**-3.48**	**<0.0001**
Symbiont (present)	0.96	0.51	1.89	0.058	**2.43**	**0.71**	**3.44**	**0.001**	**-1.35**	**0.60**	**-2.24**	**0.025**
Week	**-0.40**	**0.20**	**-1.99**	**0.047**	0.12	0.23	0.53	0.597	**0.44**	**0.20**	**2.23**	**0.026**
Week 2	**0.04**	**0.02**	**2.41**	**0.016**	-0.02	0.02	-0.83	0.406	**-0.03**	**0.01**	**-2.18**	**0.029**
Symbiont x Week					**-0.81**	**0.12**	**-6.99**	**<0.0001**				

Non-protective clone

Parameters	*Acyrthosiphon pisum*				*Megoura viciae*				*Aphis fabae*			
	Estimate	Std	z-value	p	Estimate	Std	z-value	p	Estimate	Std	z-value	p
Intercept	-1.34	0.77	-1.74	0.082	-0.89	0.68	-1.32	0.187	**-1.48**	**0.67**	**-2.21**	**0.027**
Symbiont (present)	1.06	0.88	1.21	0.227	**-1.13**	**0.58**	**-1.97**	**0.049**	**1.27**	**0.53**	**2.38**	**0.017**
Week	0.55	0.28	1.92	0.055	0.34	0.24	1.41	0.158	-0.20	0.27	-0.77	0.442
Week 2	**-0.07**	**0.02**	**-3.37**	**0.001**	**-0.09**	**0.02**	**-4.28**	**<0.0001**	**0.07**	**0.02**	**3.29**	**0.001**
Symbiont x Week	**-0.61**	**0.23**	**-2.64**	**0.008**								

Clonal lineage

Parameters	*Acyrthosiphon pisum*				*Megoura viciae*				*Aphis fabae*			
	Estimate	Std	z-value	p	Estimate	Std	z-value	p	Estimate	Std	z-value	p
Intercept	0.48	0.43	1.128	0.259	**-2.24**	**0.67**	**-3.34**	**0.001**	**-1.73**	**0.45**	**-3.83**	**<0.0001**
Clone (slow-growing)	**-0.74**	**0.31**	**-2.378**	**0.017**	**2.55**	**0.71**	**3.57**	**<0.0001**	-0.32	0.38	-0.85	0.393
Week	-0.07	0.16	-0.441	0.659	-0.03	0.20	-0.16	0.870	0.20	0.17	1.17	0.242
Week 2	-0.01	0.01	-0.564	0.573	0.00	0.01	-0.13	0.894	0.00	0.01	0.19	0.853
Symbiont x Week					**-0.36**	**0.14**	**-2.48**	**0.013**				

Table S3

Paramenters		*Acyrtosiphon pisum*		*Megoura viciae*		*Aphis fabae*	
Aphids	d.f. *	F-value	p	F-value	p	F-value	p
Intercept	1, 398	**568.93**	**<.0001**	**336.83**	**<.0001**	**709.43**	**<.0001**
Symbiont	1, 27	1.61	0.216	**9.70**	**0.004**	0.18	0.677
Clone	1, 27	**58.06**	**<.0001**	0.11	0.919	1.61	0.215
Week	1, 398	**72.06**	**<.0001**	**224.57**	**<.0001**	**17.24**	**<.0001**
Week 2	1, 398	**13.57**	**0.000**	0.77	0.381	1.53	0.217
Symbiont x Clone	1, 27	**10.89**	**0.002**			**15.54**	**0.001**

		Aphidius ervi		*Aphidius megourae*		*Lysiphlebus fabarum*	
Parasitoids	d.f. **	F-value	p	F-value	p	F-value	p
Intercept	1, 398	**578.61**	**<.0001**	**288.41**	**<.0001**	**172.60**	**<.0001**
Symbiont	1, 27	**84.85**	**<.0001**	**9.89**	**0.004**	0.30	0.587
Clone	1, 27	**14.72**	**<.0001**	0.54	0.470	**9.08**	**0.006**
Week	1, 398	**89.77**	**<.0001**	**317.46**	**<.0001**	0.27	0.605
Week 2	1, 398	**3.08**	**<.0001**	**0.03**	**0.868**	1.96	0.163
Symbiont x Clone	1, 27	**79.07**	**<.0001**			**14.76**	**0.001**

Figure S4

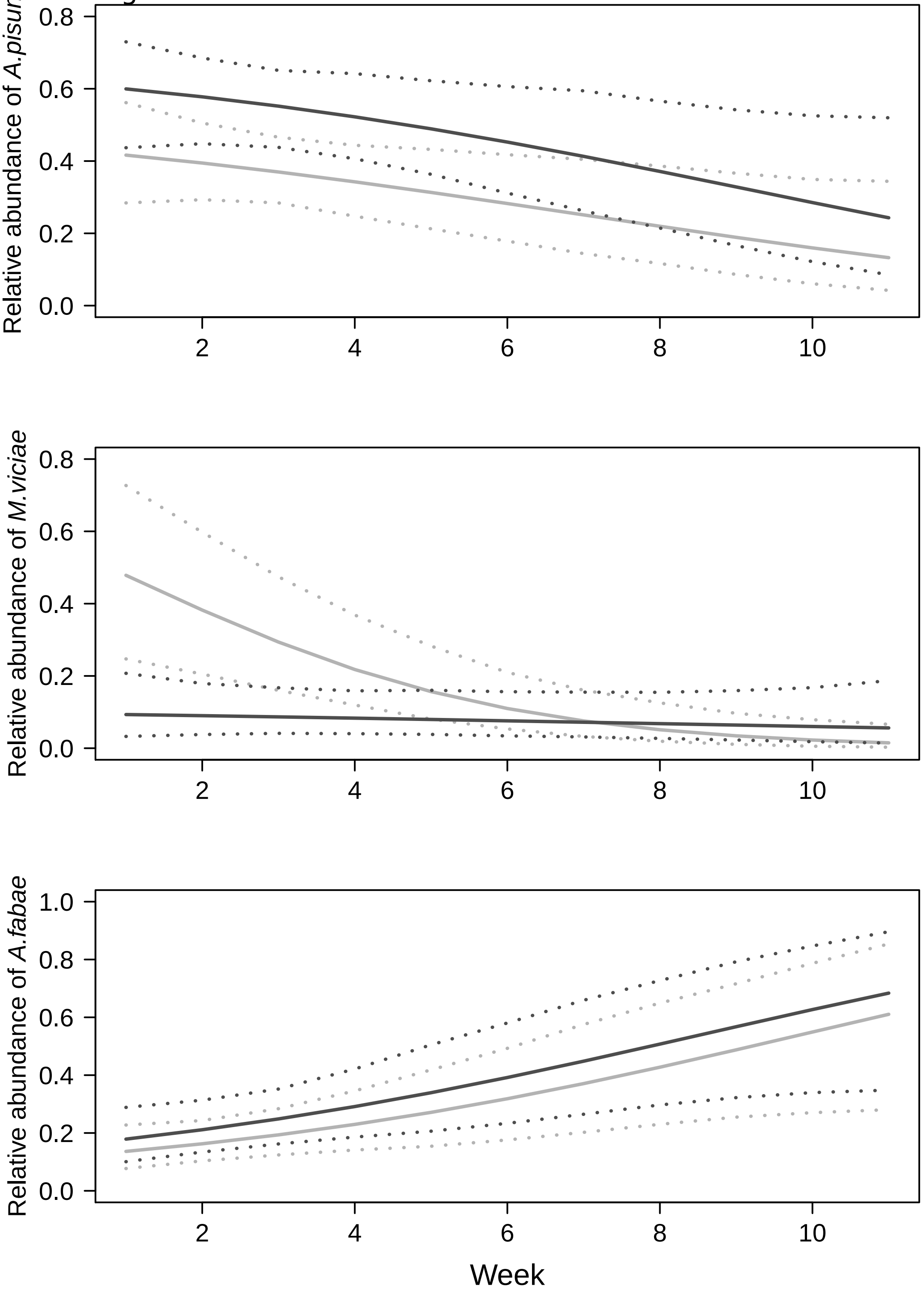

Relative abundance of *A.pisum*
0.0
0.2
0.4
0.6
0.8
2
4
6
8
10
Relative abundance of *M.viciae*
0.0
0.2
0.4
0.6
0.8
2
4
6
8
10
Relative abundance of *A.fabae*
0.0
0.2
0.4
0.6
0.8
1.0
2
4
6
8
10
Week

Figure S5

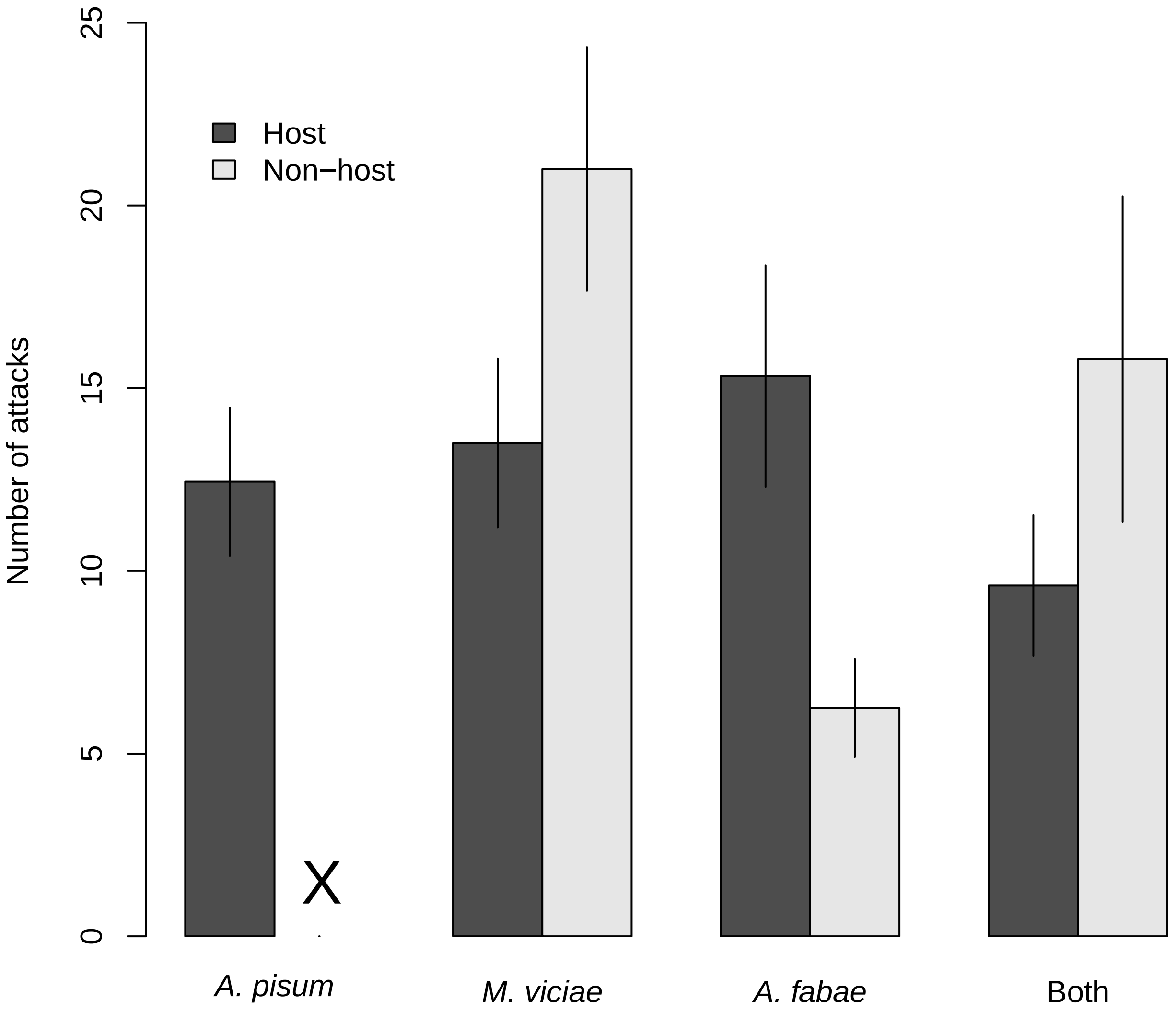

25
20
15
10
5
0
Number of attacks
Host
Non−host
X
A. pisum
M. viciae
A. fabae
Both
